# A Modified TurboEdit Cycle-Slip Detection and Correction Method for Dual-Frequency Smartphone GNSS Observation

**DOI:** 10.3390/s20205756

**Published:** 2020-10-10

**Authors:** Xiaofei Xu, Zhixi Nie, Zhenjie Wang, Yuanfan Zhang

**Affiliations:** College of Oceanography and Space Informatics, China University of Petroleum, Qingdao 266580, China; xuxiaofei.qd@gmail.com (X.X.); sdwzj@upc.edu.cn (Z.W.); z18010038@s.upc.edu.cn (Y.Z.)

**Keywords:** smartphone, cycle-slip, TurboEdit, epoch-differenced, wide-lane combination

## Abstract

Recently, some smartphone manufacturers have subsequently released dual-frequency GNSS smartphones. With dual-frequency observations, the positioning performance is expected to be significantly improved. Cycle-slip detection and correction play an important role in high-precision GNSS positioning, such as precise point positioning (PPP) and real-time kinematic (RTK) positioning. The TurboEdit method utilizes Melbourne–Wübbena (MW) and phase ionospheric residual (PIR) combinations to detect cycle-slips, and it is widely used in the data processing applications for geodetic GNSS receivers. The smartphone pseudorange observations are proved to be much noisier than those collected with geodetic GNSS receivers. Due to the poor pseudorange observation, the MW combination would be difficult to detect small cycle-slips. In addition, some specific cycle-slip combinations, where the ratio of cycle-slip values at different carrier frequencies is close to the frequency ratio, are also difficult to be detected by PIR combination. As a consequence, the traditional TurboEdit method may fail to detect specific small cycle-slip combinations. In this contribution, we develop a modified TurboEdit cycle-slip detection and correction method for dual-frequency smartphone GNSS observations. At first, MW and PIR combinations are adopted to detect cycle-slips by comparing these two combinations with moving-window average values. Then, the epoch-differenced wide-lane combinations are used to estimate the changes of smartphone position and clock bias, and the cycle-slip is identified by checking the largest normalized residual whether it exceeds a predefined threshold value. The process of estimation and cycle-slip identification is implemented in an iterative way until there is no over-limit residual or there is no redundant measurement. At last, the cycle-slip values at each frequency are estimated with the epoch-differenced wide-lane and ionosphere-free combinations, and the least-square ambiguity decorrelation adjustment (LAMBDA) method is adopted to further obtain an integer solution. The proposed method has been verified with 1 Hz dual-frequency smartphone GNSS data. The results show that the modified TurboEdit method can effectively detect and correct even for specific small cycle-slip combinations, e.g., (4, 3), which is difficult to be detected with the traditional TurboEdit method.

## 1. Introduction

In May 2016, Google released an application programming interface (API), which makes the developers access global navigation satellite system (GNSS) raw measurements, including pseudorange, phase, and doppler measurements, in Android 7.0 and up [1]. In the past years, single-frequency GNSS chipsets are dominant in the smartphone market. With a large noise level of smartphone single-frequency GNSS measurements, the positioning accuracy and reliability are thus unsatisfactory, especially under complex conditions [2,3].

Innovatively, in May 2018, the world’s first dual-frequency GNSS smartphone produced by Xiaomi was launched. It is equipped with a Broadcom BCM47755 chipset, which is a dual-frequency (L1/L5+E1/E5a) GNSS chipset [4,5,6]. Subsequently, some smartphone manufacturers have recently released dual-frequency GNSS smartphones, e.g., P30/P30 Pro from Huawei, Galaxy Note 10/10+ from Samsung, One Plus 7 from OnePlus, etc., (https://developer.android.com). A series of data quality assessment and positioning algorithms based on the smartphone dual-frequency GNSS observations were carried out [5,6,7,8,9,10]. According to these published researches, the positioning performance with smartphone dual-frequency GNSS observations has been improved effectively thanks to the newly added L5/E5a signals. In GNSS high-precision positioning, such as real-time kinematic (RTK) positioning and precise point positioning (PPP), the GNSS phase measurements are necessarily applied, and they are desired to be continuously tracked. However, the discontinuities of GNSS phase observations, widely called cycle-slips, usually happen due to signal obstacles, large environment noise, strong electromagnetic interference, etc., [11]. Therefore, cycle-slip detection and correction are very crucial for phased-based GNSS high-precision positioning.

As an important and challenging issue in GNSS data preprocessing, lots of algorithms have been proposed for cycle-slip detection and correction in the past decades. These methods include phase ionospheric residual method [12], Kalman filtering based method [13], polynomial fitting method [14,15], high-order difference method [16], and wavelet transform method [17,18]. Each of these methods has its own limitations [19]. The TurboEdit algorithm, initially developed by Blewitt (1990), is an easy and effective cycle-slip detection method [20,21]. In addition, it has been widely applied in the high-precision GNSS data processing software, such as GIPSY, PANDA, and Bernese 5.x [22,23,24]. The TurboEdit algorithm utilizes Melbourne–Wübbena (MW) combination and phase ionospheric residual (PIR) combination to detect cycle-slips. The PIR combination is insensitive to special cycle-slips where the ratio of cycle-slip values at each carrier frequency is close to the frequency ratio, but the MW combination can benefit by detecting these special cycle-slips. However, the efficiency of detecting cycle-slips with the MW combination will decrease when applying it to the smartphone GNSS measurements due to its poor pseudorange. Miao et al. (2011) proposed a modified TurboEdit method for satellite-borne GPS observations by introducing a satellite elevation weighted factor to MW combination [19]. However, this method does not change the situation that the noise of MW combination is too large to detect small cycle-slips for smartphone GNSS measurements. In order to reduce the effect of the noise of MW combination, Cai et al. (2013) applied a forward and backward moving window averaging algorithm. Obviously, this method is only suitable for GNSS data post-processing [25]. In a word, the TurboEdit method needs to be advanced for cycle-slip detection of smartphone GNSS observations.

We propose a modified TurboEdit cycle-slip detection and correction method for dual-frequency smartphone GNSS observations. At first, the MW and PIR combinations are adopted to detect cycle-slips by comparing these two combinations with moving-window average values. Most of the cycle-slips are expected to be detected in this step. Then, the epoch-differenced wide-lane combinations are used to estimate the changes of smartphone position and clock bias by applying least-square adjustment. The largest normalized residual is sorted out and checked whether it exceeds the threshold value. If the largest normalized residual is bigger than the predefined threshold, both dual-frequency phase observations of the corresponding satellite are marked as cycle-slip. The process of estimation and cycle-slip identification is implemented in an iterative way until there is no over-limit residual or there is no redundant measurement. At last, the float cycle-slip values at two frequencies can be estimated by using the epoch-differenced wide-lane and ionosphere-free combinations, and then the least-square ambiguity decorrelation adjustment (LAMBDA) method is applied to obtain an integer solution of cycle-slips.

The rest of this paper is organized as follows. In Section 2, the modified TurboEdit cycle-slip detection and correction method is discussed in detail. In Section 3, the performance is evaluated with the collected dual-frequency smartphone GNSS measurements. Finally, conclusions are summarized in the last section.

## 2. Methodology

### 2.1. Traditional TurboEdit Algorithm

The GNSS raw code and phase measurements read as [26]:

(1) $$\{\begin{array}{c} P_{i}=\rho +c\left(dt_{r}-dt^{s}\right)+trop+ion+\varepsilon_{P_{i}}\\ L_{i}=\rho +c\left(dt_{r}-dt^{s}\right)+trop-ion+\lambda_{i}N_{i}+\varepsilon_{L_{i}} \end{array}$$

where $P_{i}$, $L_{i}$ denote code and phase measurements; the subscript i represents the carrier frequency; ρ is the geometric distance between receiver and satellite; $dt_{r}$, $dt^{s}$ are the clock offsets at the receiver and satellite; trop, ion represent the tropospheric and ionospheric delays; c, $\lambda_{i}$ are the speed of light and the wavelength, $N_{i}$ is the ambiguity; $\varepsilon_{P_{i}}$, $\varepsilon_{L_{i}}$ denote the code and phase measurement noises including multipath errors.

In the traditional TurboEdit method, the cycle-slip detection is based on MW and PIR combinations. With dual-frequency smartphone GNSS observations, these two combinations can be formed as follows [27]:

(2) $$\{\begin{array}{c} L_{MW}=\frac{f_{1}L_{1}-f_{5}L_{5}}{f_{1}-f_{5}}-\frac{f_{1}P_{1}+f_{5}P_{5}}{f_{1}+f_{5}}=\lambda_{WL}N_{WL}\\ L_{PIR}=L_{1}-L_{5}=\lambda_{1}N_{1}-\lambda_{5}N_{5}+\frac{f_{1}{}^{2}-f_{5}{}^{2}}{f_{5}{}^{2}}ion \end{array}$$

where $L_{MW}$,$\;L_{PIR}$ represent the MW and PIR combinations, respectively; $f_{1}$ and $f_{5}$ are the carrier frequencies; $\lambda_{WL}=c/\left(f_{1}-f_{5}\right)$ and $N_{WL}=N_{1}-N_{5}$ denote the wide-lane wavelength and wide-lane ambiguity.

To detect cycle-slips with MW and PIR combinations, a moving-window averaging filter is utilized to calculate the average and standard deviation (STD) values:

(3) $$\{\begin{array}{c} {\bar{N}}_{WL}\left(k\right)={\bar{N}}_{WL}\left(k-1\right)+\frac{1}{k}\left[N_{WL}\left(k\right)-{\bar{N}}_{WL}\left(k-1\right)\right]\\ \sigma_{N_{WL}}^{2}\left(k\right)=\sigma_{N_{WL}}^{2}\left(k-1\right)+\frac{1}{k}\left[{\left(N_{WL}\left(k\right)-{\bar{N}}_{WL}\left(k-1\right)\right)}^{2}-\sigma_{N_{WL}}^{2}\left(k-1\right)\right] \end{array}$$

(4) $$\{\begin{array}{c} {\bar{L}}_{PIR}\left(k\right)={\bar{L}}_{PIR}\left(k-1\right)+\frac{1}{k}\left[L_{PIR}\left(k\right)-{\bar{L}}_{PIR}\left(k-1\right)\right]\\ \sigma_{L_{PIR}}^{2}\left(k\right)=\sigma_{L_{PIR}}^{2}\left(k-1\right)+\frac{1}{k}\left[{\left(L_{PIR}\left(k\right)-{\bar{L}}_{PIR}\left(k-1\right)\right)}^{2}-\sigma_{L_{PIR}}^{2}\left(k-1\right)\right] \end{array}$$

where ${\bar{N}}_{WL}$, ${\bar{L}}_{PIR}$ are the average value of $N_{WL}$ and $L_{PIR}$ in the moving-window with a window size of m; k and k−1 denote the present and previous epochs; $\sigma_{N_{WL}}$, $\sigma_{L_{PIR}}$ represent the STD of $N_{WL}$ and $L_{PIR}$, respectively.

The satellite will be marked as cycle-slip when any one of the following conditions is satisfied:

(5) $$\{\begin{array}{c} \left|N_{WL}\left(k\right)-{\bar{N}}_{WL}\left(k-1\right)\right|\geq 4\sigma_{N_{WL}}\\ \left|L_{PIR}\left(k\right)-{\bar{L}}_{PIR}\left(k-1\right)\right|\geq 4\sigma_{L_{PIR}} \end{array}$$

The noise level of smartphone pseudorange observations is much larger than that of pseudorange observations from geodetic GNSS receivers. This will lead to a larger noise level of MW combination. $\sigma_{N_{WL}}$ can be approximatively calculated in the following equation:

(6) $$\sigma_{N_{WL}}\approx \frac{\sqrt{f_{1}^{2}+f_{5}^{2}}}{f_{1}+f_{5}}\sigma_{P}\cdot \frac{1}{\lambda_{WL}}$$

At present, the noise of smartphone pseudorange observations is generally higher than 1.5 m [8], $4\cdot \sigma_{N_{WL}\;}\approx \;5.70$ cycles. Therefore, it is difficult to detect wide-lane cycle-slips of 1–5 cycles with MW combination. According to Equation (2), when the cycle-slip combination $\left(\Delta N_{1},\Delta N_{5}\right)$ nearly meets the relationship of $\Delta N_{1}/\Delta N_{5}=f_{1}/f_{5}$, it cannot be detected by PIR combination. As a result, the traditional TurboEdit method may fail to detect specific small cycle-slip combinations, e.g., (4·k, 3·k), k<6.

### 2.2. Modified TurboEdit Method

Except for specific small cycle-slip combinations, most of the cycle-slips can be detected with the traditional TurboEdit method. Therefore, the traditional TurboEdit method is first applied in our modified TurboEdit method. In order to further detect specific small cycle-slip combinations, the epoch-differenced wide-lane combinations are used to estimate the changes of smartphone position and clock bias, and the cycle-slip is identified by checking the largest normalized residual whether it exceeds the predefined threshold value. The wide-lane combination can be written as:

(7) $$L_{WL}=\frac{f_{1}L_{1}-f_{5}L_{5}}{f_{1}-f_{5}}=\rho +c\left(dt_{r}-dt^{s}\right)+trop-ion+\lambda_{WL}N_{WL}+\varepsilon_{L_{WL}}$$

where $\varepsilon_{L_{WL}}=\left(f_{1}\cdot \varepsilon_{L_{1}}-f_{5}\cdot \varepsilon_{L_{5}}\right)/\left(f_{1}-f_{5}\right)$ denotes measurement noise of the wide-lane combination. By differencing high-rate smartphone GNSS wide-lane observations at two successive epochs, the tropospheric and ionospheric delays can be largely reduced, even completely eliminated. Hence, the epoch-differenced wide-lane combination can be expressed as:

(8) $$\Delta L_{WL}=L_{WL}\left(k\right)-L_{WL}\left(k-1\right)=\Delta \rho +c\left(\Delta dt_{r}-\Delta dt^{s}\right)+\lambda_{WL}\Delta N_{WL}+\varepsilon_{\cdot L_{WL}}$$

where Δ stands for the epoch-differenced operator. The variation of wide-lane ambiguity $\Delta N_{WL}$ is expected to zero for continuous phase observations. The satellite position and clock offset can be calculated with broadcast ephemeris. Hence, the unknown parameters remain the changes of smartphone position and clock bias. All epoch-differenced wide-lane combinations, of which satellites are not marked as cycle-slip with the traditional TurboEdit method, are adopted to estimate the unknown parameters. The measurement equation can be expressed as:

(9) $$y_{WL}=A\cdot x+\varepsilon_{WL}$$

where $y_{WL}$ is the observed-minus-computed epoch-differenced wide-lane observation residual vector; A is the design matrix of the unknown parameters; $x=\left[\Delta dt_{r}\right]$ is the unknown parameter vector for the smartphone at a static scenario; and $x=\left[\Delta x_{r}\;\Delta y_{r}\;\Delta z_{r}\;\Delta dt_{r}\right]$ for the smartphone at a kinematic scenario. $\varepsilon_{WL}$ is the measurement error vector and its covariance matrix is Q. Assuming that the phase observations at two frequencies are independent at the same noise level with a zenith STD of $\sigma_{L}$, the covariance matrix Q can be derived as:

(10) $$Q=\left(\begin{array}{ccc} 2\left(\alpha_{WL}^{2}+\beta_{WL}^{2}\right)\sigma_{L}^{2}/sin^{2}\left(el_{1}\right) & \cdots & 0\\ \vdots & \ddots & \vdots \\ 0 & \cdots & 2\left(\alpha_{WL}^{2}+\beta_{WL}^{2}\right)\sigma_{L}^{2}/sin^{2}\left(el_{n}\right) \end{array}\right)$$

Here, $\alpha_{WL}=f_{1}/\left(f_{1}-f_{5}\right)$, $\beta_{WL}=-f_{5}/\left(f_{1}-f_{5}\right)$; el represents the satellite elevation with respect to the smartphone. The least-square adjustment can be used to estimate the changes of smartphone position and clock bias:

(11) $$\hat{x}={\left(A^{T}PA\right)}^{-1}A^{T}P\cdot y_{WL}$$

where $P=Q^{-1}$ denotes the weight matrix. The corrected residuals can be computed as:

(12) $$v=y_{WL}-A\cdot \hat{x}$$

Assuming that the number of observed satellites is n and the number of unknown parameters is m, the formal STD can be calculated as:

(13) $${\hat{\sigma }}_{0}=\sqrt{\frac{v^{T}Pv}{n-m-1}}$$

The cycle-slips will be identified by checking the largest normalized residual whether it exceeds a predefined threshold [28,29]:

(14) $$max\left(\frac{\left|{\hat{v}}_{k}\right|}{\sigma_{{\hat{v}}_{k}}}\right)>\eta$$

where ${\hat{v}}_{k}$ represents the k^th^ corrected residual; $\sigma_{{\hat{v}}_{k}}={\hat{\sigma }}_{0}\cdot \sqrt{Q\left[k\right]\left[k\right]}\;$ and Q[k][k] denotes k^th^ diagonal element of Q; η is the predefined threshold value. If that is the case, the satellite with the largest normalized residual will be then isolated from the least-square adjustment. An iterative way is implemented until the condition of Equation (14) is not satisfied or there is no redundancy.

Once all possible cycle-slips are detected, the cycle-slip values at each frequency will be estimated with all epoch-differenced wide-lane and ionosphere-free combinations including both cycle-slip and non-cycle-slip observations, and an integer estimation of cycle-slip values can be resolved with the LAMBDA method.

In case that the cycle-slip has been detected for a specific satellite, the estimation parameters for cycle-slip values should be introduced to the corresponding measurement equation. As a result, the unknown parameters are the changes of smartphone position, clock bias, and cycle-slip parameters. Hence, the measurement equation formed by the epoch-differenced wide-lane observations can be written as:

(15) $$y_{WL}=A_{WL}x+B_{WL}\Delta N+\varepsilon_{WL}$$

where x represents the vector composed by the change of smartphone position and clock bias; ΔN is the cycle-slip vector; $A_{WL}$ and $B_{WL}$ are the corresponding design matrices; $\varepsilon_{WL}$ is the measurement error vector.

The ionosphere-free combination formed by the carrier phase measurement at two frequencies can be expressed as [30,31]:

(16) $$L_{IF}=\alpha L_{1}+\left(1-\alpha \right)L_{5}=\rho +c\left(dt_{r}-dt^{s}\right)+trop+\lambda_{IF}N_{IF}+\varepsilon_{L_{IF}}$$

where $\alpha =f_{1}^{2}/\left(f_{1}^{2}-f_{5}^{2}\right)$; $\lambda_{IF}N_{IF}=\alpha \cdot \lambda_{1}N_{1}+\left(1-\alpha \right)\cdot \lambda_{5}N_{5}$ stands for the ionosphere-free ambiguity in meters; $\varepsilon_{L_{IF}}=\alpha \cdot \varepsilon_{L_{1}}+\left(1-\alpha \right)\cdot \varepsilon_{L_{5}}$ denotes measurement noise of the ionosphere-free phase combination. Once the epoch-differencing is employed to high-rate smartphone GNSS ionosphere-free observations, the tropospheric delay would be nearly eliminated. Consequently, the epoch-differenced ionosphere-free observation can be written as:

(17) $$\Delta L_{IF}=L_{IF}\left(k\right)-L_{IF}\left(k-1\right)=\Delta \rho +c\left(\Delta dt_{r}-\Delta dt^{s}\right)+\lambda_{IF}\Delta N_{IF}+\varepsilon_{\Delta L_{IF}}$$

Broadcast ephemeris can be adopted to calculate the satellite position and clock offset. Similarly, all epoch-differenced ionosphere-free observations at the current epoch are employed to estimate the unknown parameters. The measurement equation can be expressed as:

(18) $$y_{IF}=A_{IF}x+B_{IF}\Delta N+\varepsilon_{IF}$$

where $A_{IF}$ and $B_{IF}$ are the design matrices with respect to x and ΔN; $\varepsilon_{IF}$ is the measurement error vector of ionosphere-free phase combination. Combining Equations (15) and (18), we yield:

(19) $$\left(\begin{array}{c} y_{WL}\\ y_{IF} \end{array}\right)=\left(\begin{array}{c} A_{WL}\\ A_{IF} \end{array}\right)\cdot x+\left(\begin{array}{c} B_{WL}\\ B_{IF} \end{array}\right)\cdot \Delta N+\left(\begin{array}{c} \varepsilon_{WL}\\ \varepsilon_{IF} \end{array}\right)$$

The float estimation of cycle-slips can be calculated by applying the least-square adjustment to Equation (19). With float estimation of cycle-slips, the LAMBDA technique can be employed to obtain a integer solution of cycle-slips [32]. Furthermore, the integer estimation of cycle-slips is validated by applying the ratio test [33,34].

**Figure 1:**
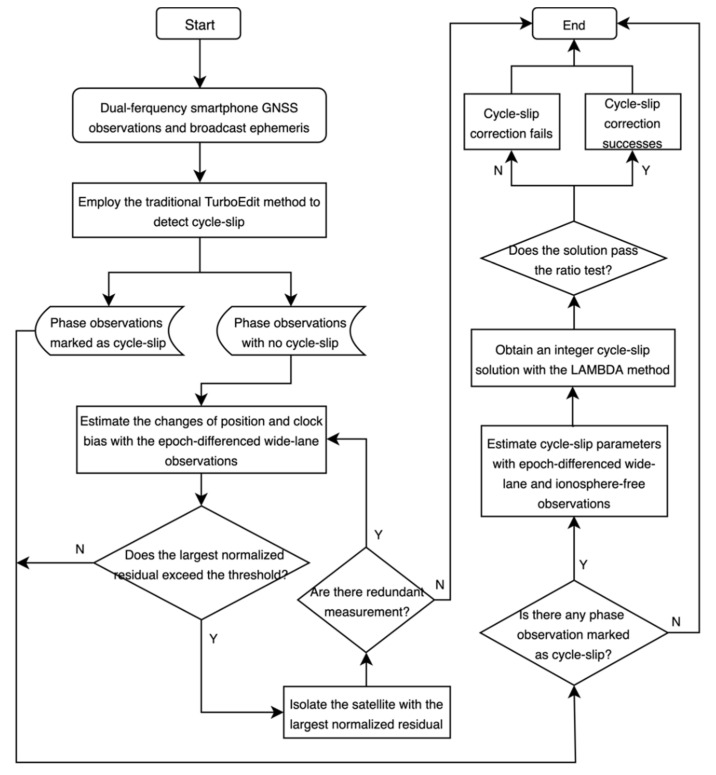
Procedure of the modified TurboEdit method for dual-frequency smartphone global navigation satellite system (GNSS) observations.

The procedure of the modified TurboEdit method is shown as Figure 1. At first, the MW and PIR combinations are used to detect the cycle-slip satellite by a satellite. Most of the cycle-slips will be detected in this step except for some specific small cycle-slip combinations. Then, the epoch-differenced wide-lane combination is applied to estimate the changes of position and clock bias. The cycle-slip is identified by checking the largest normalized residual whether it exceeds the predefined threshold value. If yes, the satellite with the largest normalized residual will be isolated from the least-square adjustment. The process of estimation and cycle-slip identification is implemented in an iterative way until there is no over-limit residual or there is no redundant measurement. Finally, the cycle-slip values at each frequency are estimated with the epoch-differenced wide-lane and ionosphere-free combinations, and the LAMBDA method is adopted to further obtain an integer cycle-slip solution.

## 3. Experimental Results and Analysis

**Figure 2:**
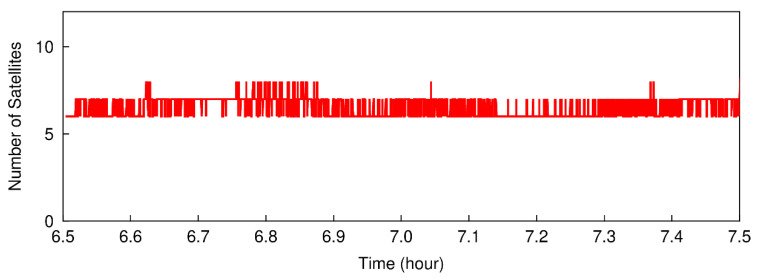
The number of visible GPS and Galileo satellites with dual-frequency observations.

In this section, the performance of the modified TurboEdit method will be presented by using the real dual-frequency smartphone GNSS data. The raw GNSS dataset, including GPS L1/L5 and Galileo E1/E5a code and phase measurements, was collected with a Xiaomi Mi-8 smartphone under static condition from 06:40:00 to 07:30:00, on August 20, 2019, in GPS time. The GNSS observations were recorded at a sample interval of 1 s. Two data subsets, from 06:42:00 to 06:46:59 and from 06:50:00 to 06:54:59, are extracted from the collected dataset. One is used to analyze the performance of detecting and correcting small cycle-slips, and the other is used to verify the effectiveness of detecting and correcting specific small cycle-slip combinations. These two data subsets are assumed to be collected in kinematic scenes during the following process of cycle-slip detection and correction. The cutoff elevation is set to 7 degrees. Figure 2 shows the number of visible GPS and Galileo satellites with dual-frequency observations.

### 3.1. Small Cycle-Slip Combination Detection and Correction

**Figure 3:**
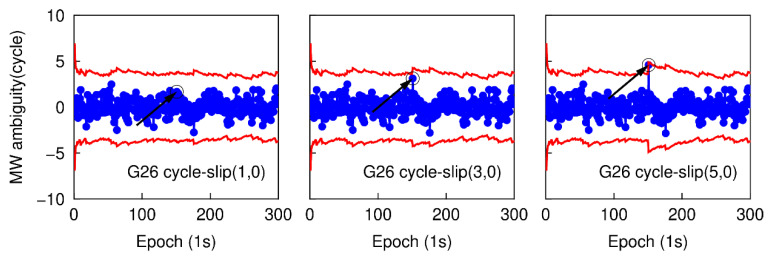
Small cycle-slip detection results using Melbourne–Wübbena (MW) combination for G26.

**Figure 4:**
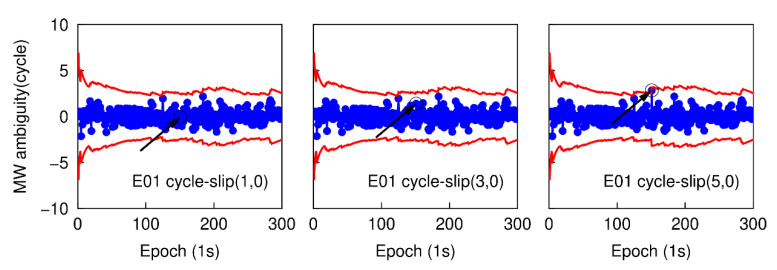
Small cycle-slip detection results using MW combination for E01.

**Figure 5:**
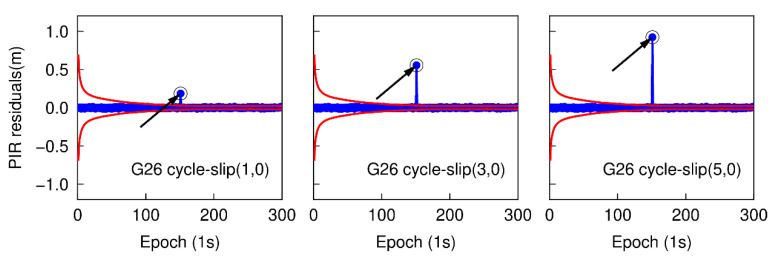
Small cycle-slip detection results using phase ionospheric residual (PIR) combination for G26.

**Figure 6:**
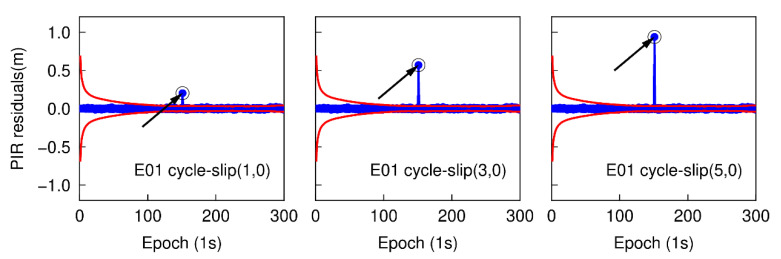
Small cycle-slip detection results using PIR combination for E01.

Using the first data subset, six cases are simulated to analyze the performance of detecting small cycle-slips. In each case, the simulated cycle-slip is added to raw GNSS phase observations manually. These six simulated small cycle-slip cases include 1, 3, 5 cycles on the G26 L1 carrier phase signal at the 151th epoch, and the same cycle-slips on the E01 E1 carrier phase signal at the same epoch. The initial STD values of $N_{WL}$ and $L_{PIR}$ are set to 3.0 cycles and 0.028 m, respectively, which are approximately calculated according to the noise level of smartphone measurements at present [8]. The time series of $N_{WL}\left(k\right)-{\bar{N}}_{WL}\left(k-1\right)$ in six different cases are shown in Figure 3 and Figure 4. The blue points represent the variation of $N_{WL}\left(k\right)-{\bar{N}}_{WL}\left(k-1\right)$ and the red curves denote the cycle-slip boundaries formed by $\pm 4\sigma_{N_{WL}}$. It is clear that the variation of $N_{WL}\left(k\right)-{\bar{N}}_{WL}\left(k-1\right)$ is well bounded by the red curves. Hence, no cycle-slip is identified by MW combination according to the cycle-slip judgment condition in Equation (5). The failure to identify the small cycle-slips is because of the poor smartphone pseudorange. Shown in Figure 5 and Figure 6 are the cycle-slip detection results for the G26 and E01 by using the PIR combination. The blue points present the variation of $L_{PIR}\left(k\right)-{\bar{L}}_{PIR}\left(k-1\right)$ and red curves are the corresponding boundaries yielded by $\pm 4\sigma_{N_{PIR}}$. Obviously, the simulated cycle-slips are uniquely identified by PIR combination. Therefore, all simulated small cycle-slips can be correctly detected with the traditional TurboEdit method, and this also indicates that most of the cycle-slips can be identified by applying the traditional TurboEdit method.

**Figure 7:**
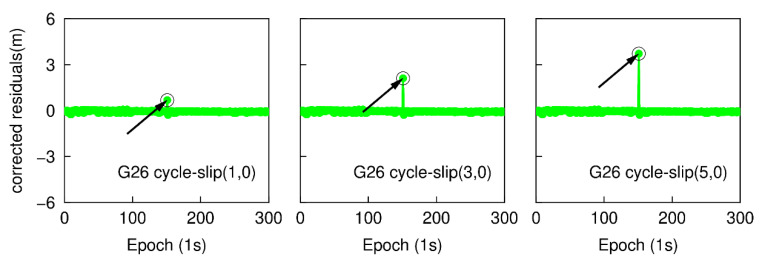
Small cycle-slip detection results by checking the largest residual for G26.

**Figure 8:**
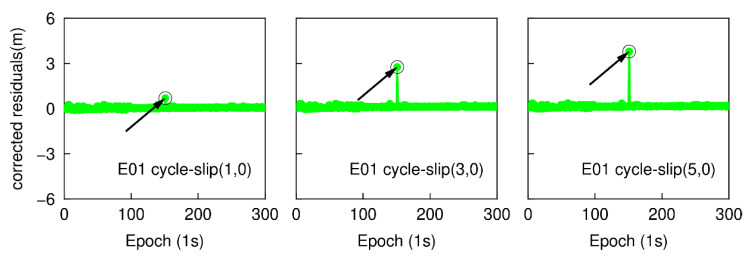
Small cycle-slip detection results by checking the largest residual for E01.

With the same GPS and Galileo dual-frequency observations as above, the epoch-differenced wide-lane combinations are employed to estimate the changes of smartphone position and clock bias, and the time series of the residuals of G25 and E01 are shown in Figure 7 and Figure 8. The green points represent the variation of the residuals. The over-limit residuals are 0.676, 2.105, 3.716 m for G26 and 0.691, 2.690, 3.681 m for E01 at the 151th epoch. Obviously, the simulated cycle-slip (1, 0), (3, 0), and (5, 0) can be accurately identified.

Once cycle-slips have been detected, all epoch-differenced wide-lane and ionosphere-free observations at the same epoch are adopted to estimate the changes of smartphone clock bias and cycle-slip parameters. The differences between the cycle-slip float estimations and the real values are 0.021, 0.018, 0.023 cycles for G26 and 0.023, 0.026, 0.023 cycles for E01. The LAMBDA method is further employed to obtain an integer solution of cycle-slips, which is presented in Table 1. The integer solution is validated by applying the ratio test with a threshold of 3.0. The ratio values are 438.12, 453.80, 408.80 for G26 and 382.60, 368.79, 395.97 for E01, respectively. Therefore, the integer solution of cycle-slip can be accepted. It is clear that by checking the largest residual of exceeding the predefined threshold can also uniquely identify the small cycle-slips (range from 1 to 5 cycles) for dual-frequency smartphone GNSS observations.

### 3.2. Specific Small Cycle-Slip Combination Detection and Correction

**Figure 9:**
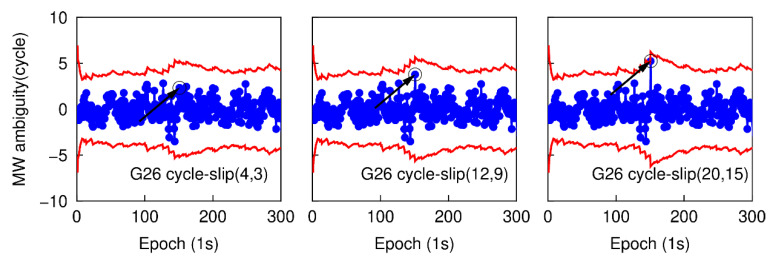
Specific small cycle-slip combination detection results with MW combination for G26.

**Figure 10:**
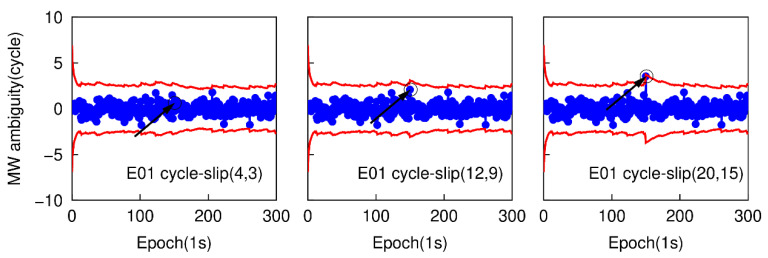
Specific small cycle-slip combination detection results with MW combination E01.

**Figure 11:**
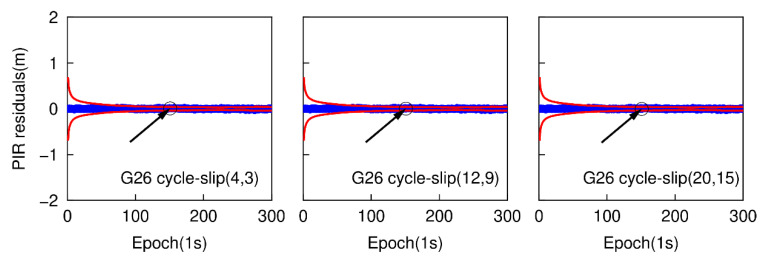
Specific small cycle-slip combination detection results with PIR combination for G26.

**Figure 12:**
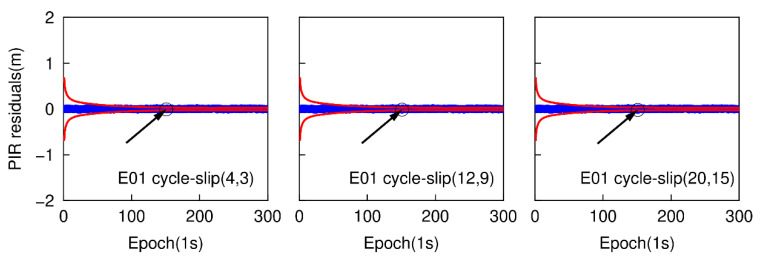
Specific small cycle-slip combination detection results with PIR combination for E01.

Most of the cycle-slips can be detected by applying the traditional TurboEdit method, but it may fail to identify some specific small cycle-slip combinations. For instance, if the cycle-slip combinations satisfy the conditions that $\left|\Delta N_{1}-\Delta N_{5}\right|$ is small than six cycles and $\left(\Delta N_{1}/\Delta N_{5}\right)\approx \left(f_{1}/f_{5}\right)$, the cycle-slip may not be identified by the traditional TurboEdit method. In this part, a group of specific small cycle-slips, which are (4, 3), (12, 9), and (20, 15) are simulated. These three specific small cycle-slip combinations are chosen because they are difficult to be detected by applying the traditional TurboEdit method alone. Based on the second data subset, six cases are also simulated by adding the cycle-slip combinations of (4, 3), (12, 9), and (20, 15) on the G26 L1 and L5 carrier phase signals at the 151th epoch, and adding the same cycle-slip combinations on the E01 E1 and E5a carrier phase signal at the same epoch. The proposed method is employed to detect and correct cycle-slip combinations with the simulated six sub-datasets. Figure 9 and Figure 10 show the time series of $N_{WL}\left(k\right)-{\bar{N}}_{WL}\left(k-1\right)$ and the cycle-slip boundaries formed by $\pm 4\sigma_{N_{WL}}$. The blue points still represent the variation of $N_{WL}\left(k\right)-{\bar{N}}_{WL}\left(k-1\right)$ and the red curves denote the corresponding boundaries. The values of $L_{PIR}\left(k\right)-{\bar{L}}_{PIR}\left(k-1\right)$ and the cycle-slip boundaries formed by $\pm 4\sigma_{N_{PIR}}$ at different epochs are shown in Figure 11 and Figure 12. It is apparent that the values of $N_{WL}\left(k\right)-{\bar{N}}_{WL}\left(k-1\right)$ and $L_{PIR}\left(k\right)-{\bar{L}}_{PIR}\left(k-1\right)$ are well constrained by the cycle-slip boundaries. In other words, both MW combination and PIR combination have failed to identify these specific small cycle-slip combinations. Therefore, we can conclude that these specific small cycle-slip combinations cannot be detected by the traditional TurboEdit method with dual-frequency smartphone GPS/Galileo observations.

**Figure 13:**
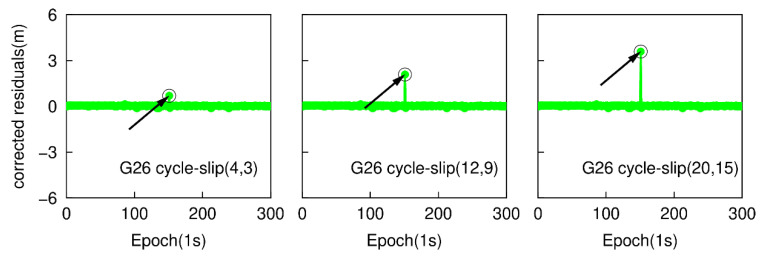
Specific small cycle-slip combination detection results by checking the largest residual for G26.

**Figure 14:**
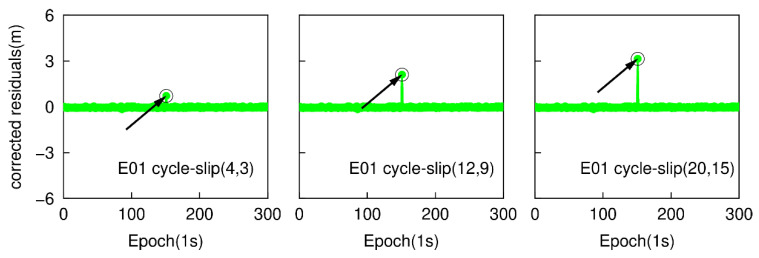
Specific small cycle-slip combinations detection results by checking the largest residual for E01.

The epoch-differenced wide-lane combinations, formed by GPS L1/ L5 and Galileo E1/E5a phase observations, are utilized to estimate the changes of smartphone positions and clock bias by applying the least-square adjustment. Figure 13 and Figure 14 show the time series of the residuals of G26 and E01. The over-limit residuals are 0.639, 2.122, 3.535 m for G26 and 0.742, 2.187, 3.227 m for E01 at the 151th epoch. That is to say, the simulated specific small cycle-slip combinations (4, 3), (12, 9), (20, 15) can be clearly detected by checking the largest normalized residual of exceeding the predefined threshold.

After all cycle-slip combinations are detected, the float cycle-slip values at each frequency can be estimated with the epoch-differenced wide-lane and ionosphere-free observations. The biases between the cycle-slip float estimations and the real values are 0.021, 0.020, 0.023 cycles for G26 and 0.020, 0.013, 0.020 cycles for E01. By further employing the LAMBDA method, an integer solution of cycle-slips can be obtained which is shown in Table 2. The ratio values are 428.82, 455.23, 361.20 for G26 and 449.13, 502.61, 438.91 for E01. Obviously, all integer solutions of cycle-slip combinations can be accepted. It illustrates that the modified TurboEdit method can correctly detect and repair the simulated cycle-slip combinations. Compared to the traditional TurboEdit method, the modified TurboEdit method is more effective in detecting specific small cycle-slip combinations for dual-frequency smartphone GNSS observations.

In summary, given the poor smartphone pseudorange observations, it is difficult to detect a specific small cycle-slip combination with the traditional TurboEdit method when the ratio of cycle-slips at each frequency is close to that of carrier frequencies. The experiment results show that all the simulated cycle-slips are successfully detected and corrected by the modified TurboEdit method, and the modified TurboEdit method is effective even for specific small cycle-slip combinations, which is difficult to be identified with the traditional TurboEdit method.

## 4. Conclusions

Cycle-slip detection and correction play an important role in high-precision GNSS positioning. It is a great challenge to detect and correct specific small cycle-slip combinations for smartphone GNSS observations. In this contribution, a modified TurboEdit method has been proposed for dual-frequency smartphone GNSS observations. In the modified TurboEdit method, MW and PIR combinations are first utilized to detect the cycle-slip. Most of the cycle-slips are expected to be detected in this step. Then, the epoch-differenced wide-lane combinations are used to estimate the changes of smartphone position and clock bias, and the cycle-slip is identified by checking the largest normalized residual whether it exceeds the predefined threshold value. The process of estimation and cycle-slip identification is implemented in an iterative way until there is no over-limit residual or there is no redundant measurement. Once all cycle-slips are detected, the float values of cycle-slips at each frequency are estimated with the epoch-differenced wide-lane and ionosphere-free combinations, and LAMBDA is further employed to obtain an integer solution of cycle-slips.

The performance of the modified TurboEdit method has been evaluated with dual-frequency smartphone GNSS observations collected by Xiaomi Mi-8. The results indicate that the modified TurboEdit method can effectively detect and correct the simulated cycle-slip combinations. Compared with the traditional TurboEdit method, the modified TurboEdit method is more effective in detecting specific small cycle-slip combinations.

## Figures and Tables

**Table 1:** Summary of the small cycle-slips detection and correction results.

Cycle-Slip Combinations $\left(\Delta N_{1},\;\Delta N_{5}\right)$	Satellite	Cycle-Slip Detectable?			The Ratio Test Values	Integer Estimation of Cycle-Slips $\left(\Delta N_{1},\;\Delta N_{5}\right)$
		MW	PIR	Modified Method		
(1, 0)	G26	No	Yes	Yes	438.12	(1, 0)
	E01	No	Yes	Yes	382.60	
(3, 0)	G26	No	Yes	Yes	453.80	(3, 0)
	E01	No	Yes	Yes	368.79	
(5, 0)	G26	No	Yes	Yes	408.80	(5, 0)
	E01	No	Yes	Yes	395.97	

**Table 2:** Summary of the specific small cycle-slips detection and correction results.

Cycle-Slip Combinations $\left(\Delta N_{1},\;\Delta N_{5}\right)$	Satellite	Cycle-Slip Detectable?			The Ratio Test Values	Integer Estimation of Cycle-Slips $\left(\Delta N_{1},\;\Delta N_{5}\right)$
		MW	PIR	Modified Method		
(4, 3)	G26	No	No	Yes	428.82	(4, 3)
	E01	No	No	Yes	449.13	
(12, 9)	G26	No	No	Yes	455.23	(12, 9)
	E01	No	No	Yes	502.61	
(20, 15)	G26	No	No	Yes	361.20	(20, 15)
	E01	No	No	Yes	438.91	
